# Correction: Convergent synthesis of thiodiazole dioxides from simple ketones and amines through an unusual nitrogen-migration mechanism

**DOI:** 10.1039/d4sc90120g

**Published:** 2024-06-24

**Authors:** Kunlayanee Punjajom, Paul P. Sinclair, Ishika Saha, Mark Seierstad, Michael K. Ameriks, Pablo García-Reynaga, Terry P. Lebold, Richmond Sarpong

**Affiliations:** a Department of Chemistry, University of California Berkeley CA 94720 USA rsarpong@berkeley.edu; b Janssen Research and Development San Diego California 92121 USA pgarciar@its.jnj.com terry.lebold@gmail.com

## Abstract

Correction for ‘Convergent synthesis of thiodiazole dioxides from simple ketones and amines through an unusual nitrogen-migration mechanism’ by Kunlayanee Punjajom *et al.*, *Chem. Sci.*, 2024, **15**, 328–335, https://doi.org/10.1039/D3SC04478E.

The original article contains errors in Scheme 1 which include: (1) the structure of **3w** in which the compound is depicted as the 5,6,7,8-tetrahydroquinoline rather than the intended tetrahydronaphthalene; (2) the superscripts for the yields of **3w**, in which the superscripts for compound **3c** were repeated. The yields should read:

9% (83%)^*d*^

33% (40%)^*e*^

These changes do not affect the conclusions of the manuscript. An updated figure and caption are included here.

**Scheme 1:**
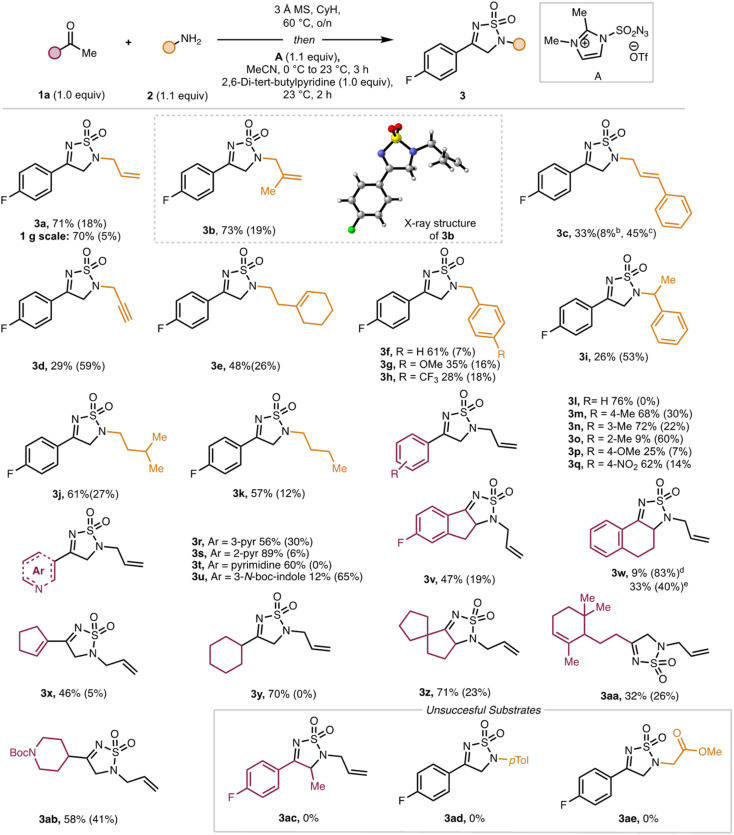
Substrate scope of amine. ^*a*^Reaction conditions: see Table 1, entry 4. ^*b*^p-Fluoroacetophenone. ^*c*^(*E*)-Cinnamylamine. ^*d*^From α-tetralone ^*e*^from β-tetralone. CyH: cyclohexane, MS: molecular sieve. Isolated yield reported. Yield in parentheses refers to recovered starting material.

In Fig. 2C, the ^15^N product should be labeled ^15^N-**3w** and not **3x**. In addition, the caption for Fig. 2 part D was missing in the original article. An updated figure and caption are provided here.

**Fig. 2:**
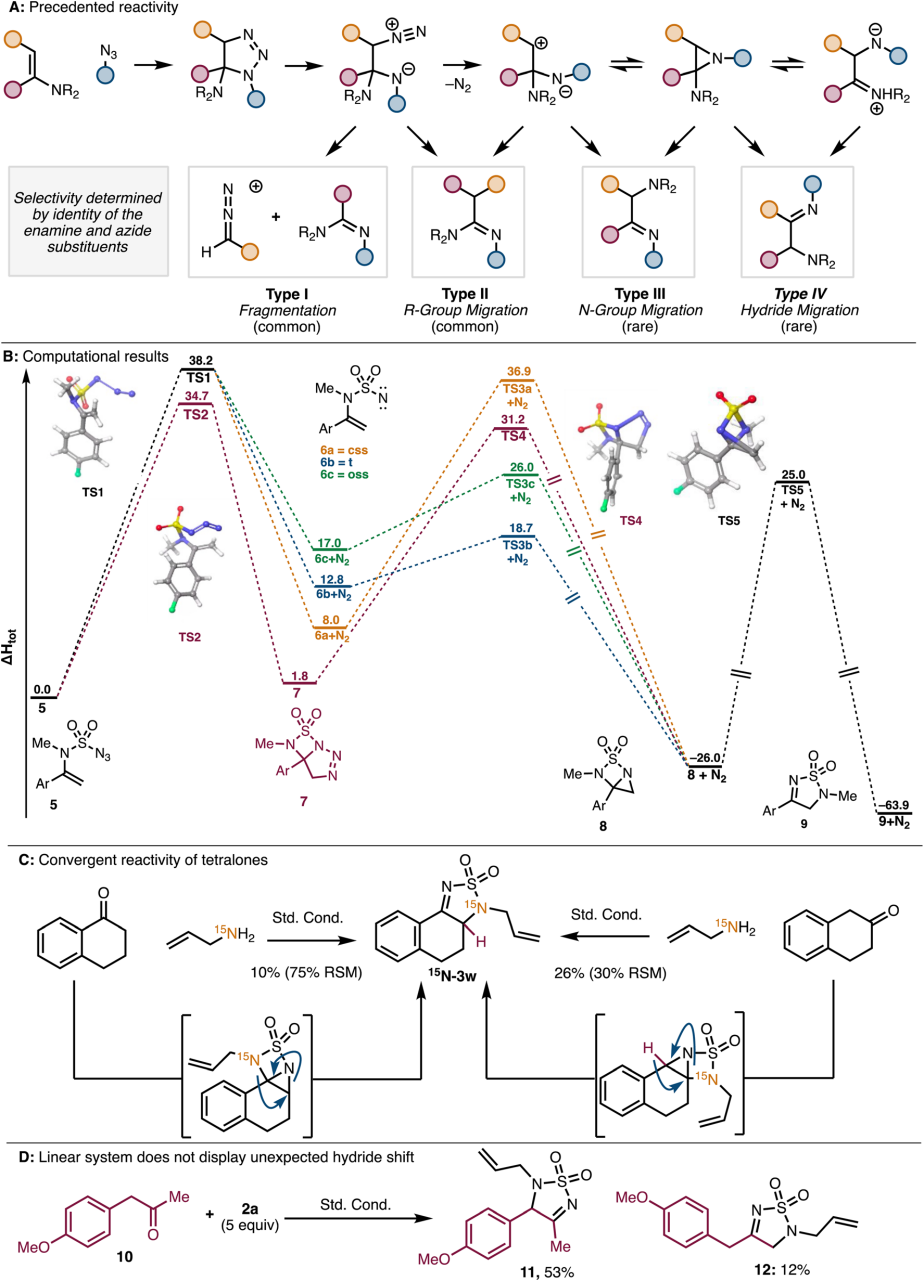
(A) Rearrangements of amino-triazolines. (B) DFT investigation of possible reaction pathways (values given in kcal mol^−1^). (C) Reactivity of tetralone substrates. (D) Reaction with homo-benzylic ketone showing no nitrogen migration. (css: closed shell singlet, oss: open shell singlet, t: triplet.).

The Royal Society of Chemistry apologises for these errors and any consequent inconvenience to authors and readers.

